# *DNMT1* and *AIM1* Imprinting in human placenta revealed through a genome-wide screen for allele-specific DNA methylation

**DOI:** 10.1186/1471-2164-14-685

**Published:** 2013-10-05

**Authors:** Radhika Das, Yew Kok Lee, Ruslan Strogantsev, Shengnan Jin, Yen Ching Lim, Poh Yong Ng, Xueqin Michelle Lin, Keefe Chng, George SH Yeo, Anne C Ferguson-Smith, Chunming Ding

**Affiliations:** 1 Singapore Institute for Clinical Sciences, Agency for Science, Technology and Research (A*STAR), Singapore, Singapore; 2 Department of Physiology, Development & Neuroscience, University of Cambridge, Cambridge, UK; 3 Department of Maternal Fetal Medicine, K.K. Women’s and Children’s Hospital, Singapore, Singapore

**Keywords:** Genomic imprinting, Placenta, Next generation sequencing, Differentially Methylated Region (DMR), *DNMT1*, *AIM1*

## Abstract

**Background:**

Genomic imprinting is an epigenetically regulated process wherein genes are expressed in a parent-of-origin specific manner. Many imprinted genes were initially identified in mice; some of these were subsequently shown not to be imprinted in humans. Such discrepancy reflects developmental, morphological and physiological differences between mouse and human tissues. This is particularly relevant for the placenta. Study of genomic imprinting thus needs to be carried out in a species and developmental stage-specific manner. We describe here a new strategy to study allele-specific DNA methylation in the human placenta for the discovery of novel imprinted genes.

**Results:**

Using this methodology, we confirmed 16 differentially methylated regions (DMRs) associated with known imprinted genes. We chose 28 genomic regions for further testing and identified two imprinted genes (*DNMT1* and *AIM1*). Both genes showed maternal allele-specific methylation and paternal allele-specific transcription. Imprinted expression for *AIM1* was conserved in the cynomolgus macaque placenta, but not in other macaque tissues or in the mouse.

**Conclusions:**

Our study indicates that while there are many genomic regions with allele-specific methylation in tissues like the placenta, only a small sub-set of them are associated with allele-specific transcription, suggesting alternative functions for such genomic regions. Nonetheless, novel tissue-specific imprinted genes remain to be discovered in humans. Their identification may help us better understand embryonic and fetal development.

## Background

Genomic imprinting is the epigenetic phenomenon wherein genes are expressed exclusively from one parental allele [1,2]. Imprinting has been reported in placental mammals, specifically, in primates, rodents, canines and ruminants. Some of these imprinted genes exhibit species-specific and spatial-temporal patterns of imprinted expression [3,4].

Selective inactivation of one parental allele can be achieved by parent-of-origin specific cytosine methylation. Germline-derived heritable differentially methylated regions (gDMRs) are established at the gamete stage [5]. Secondary differentially methylated marks are acquired after fertilization or later in life, and these are known as somatic DMRs or sDMRs [6]. Allele-specific activating or repressive histone modifications have also been implicated in regulating imprinting [7].

Since the discovery of the first imprinted gene in 1991, 73 imprinted genes have been identified in humans http://igc.otago.ac.nz/home.html while 155 imprinted genes have been reported in mice (http://www.mousebook.org/catalog.php?catalog=imprinting). In recent years, many studies using genome-wide technologies for genomic or epigenomic analyses were performed to identify novel imprinted genes. However, most had mixed success [8-16]. Whole genome and transcriptome sequencing technologies have helped identify only a small number of imprinted transcripts [8,17-22], suggesting that most imprinted genes have already been identified [23] or are tissue-specific and thus needed to be analyzed in specific cell types [8]. Only one study identified a large number of potential imprinted genes in the mouse brain [24,25], but further investigation revealed that most of these may be false positives due to artifacts from the RNA-Seq approach [26], a finding supported by more recent data [27].

Functionally, genomic imprinting is critical for proper placenta and embryo development [28-32]. Conditions such as Intra-Uterine Growth Restriction (IUGR) and pre-eclampsia as well as unsuccessful pregnancies have been correlated with abnormalities in methylation or aberrant expression of imprinted genes in the placenta [33-35]. Surprisingly, very few human or primate-specific placental imprinted genes are known so far, though interesting candidates like *RB1* (Table 1), *ZNF331* and the microRNA cluster C19MC have been discovered in recent screens [21,36,37]. A comparison between the 73 imprinted genes discovered to date in humans and the 155 reported in mice reveals that majority of this divergence is due to the multiple genes imprinted specifically in the mouse placenta [38,39], although recent data suggests that several genes were wrongly identified as showing imprinted expression in mouse placenta [18,40,41]. The imprinting difference is consistent with the biological differences between the less-invasive mouse placenta and its highly invasive human counterpart.

**Table 1:** Confirmation of known human germline differentially methylated regions

**Gene**	Germline DMR locus*	Overlapping UCSC CpG island*	**Average methylation**	**Reference**
*HYMAI/PLAGL1*	chr6:144323557-144324495	chr6:144328917-144329847	0.384	[42]
IGF2R**	chr6:160426265-160427502	chr6:160426265-160427502	0.698	[43]
*GRB10*	chr7:50849753-50850871	chr7:50849753-50850871	0.452	[44]
*SGCE/PEG10*	chr7:94284859-94286527	chr7:94284859-94286527	0.390	[45]
*MEST*	chr7:130130740-130133111	chr7:130130740-130133111	0.499	[46]
*H19/IGF2*	chr11:2020180-2022580	chr11:2019566-2019863	0.457	[47]
*KCNQ1*	chr11:2720354-2721827	chr11:2720411-2722087	0.504	[48]
*MEG3*	chr14:101272662-101277765	chr14:101290524-101290868	0.334	[49]
*SNRPN*	chr15:25,199,933-25200342	chr15:25200036-25201054	0.374	[50]
*PEG3*	chr19:57351284-57351995	chr19:57351284-57351995	0.420	[51]
*NNAT*	chr20:36148604-36150136	chr20:36148604-36150136	Not represented	[52]
*L3MBTL*	chr20:42143211-42143591	chr20:42143211-42143591	0.479	[53]
*GNAS-A*	chr20:57464132-57464622	chr20:57463653-57467739	0.191	[54]
*GNAS-B*	chr20:57426198-57430959	chr20:57426730-57427047	0.494	[55]
*RB1*	chr13:48892636–48893857	chr13:48892636-48893857	0.577	[56]
*INPP5F_V2*	chr10:121577530-121578385	chr10:121577530-121578385	0.456	[57]
*MCTS2*	chr20:30135077-30135292	chr20:30135077-30135292	0.633	[57]

*Based on UCSC genome build hg19.

**Polymorphic imprinting reported in human placenta, but DMR still present.

In this study, we used reduced representation bisulfite sequencing (RRBS) to identify partially methylated CpG islands (CGIs) in the human placental genome. We further identified candidate regions with allele-specific methylation based on calculation of methylation concordance values. We then selected 28 regions for further characterization and identified two novel imprinted genes (*DNMT1* and *AIM1*). Both genes are paternally expressed and methylated specifically on the maternal allele in the human placenta. For *AIM1*, the differential methylation is conserved in another primate, the cynomolgus macaque but not in the mouse. In conclusion, we have delineated many regions with allele-specific methylation and developed an approach for the identification of human placenta-specific imprinted genes.

## Results

### Confirmation of known germline differentially methylated regions using RRBS DNA methylation analysis

**Figure 1:**
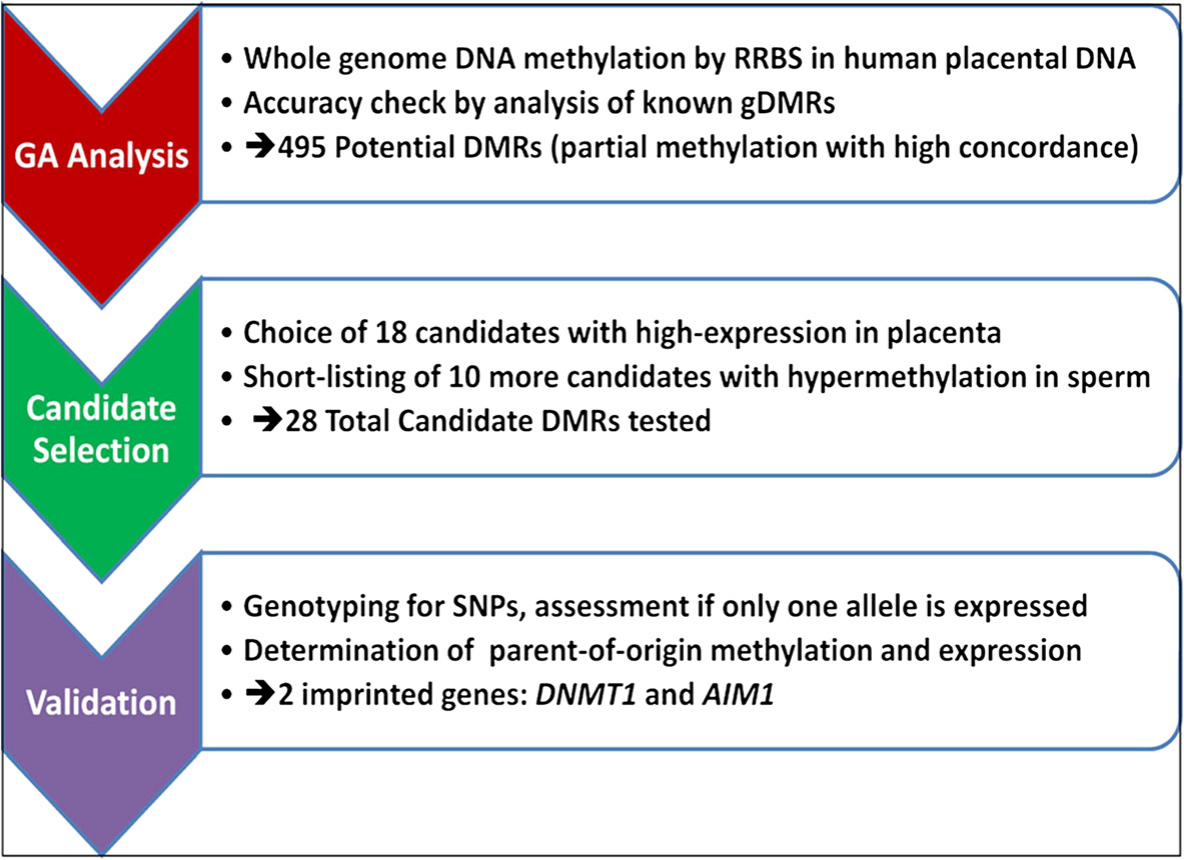
**Pipeline for assessment of allele-specific methylation and genomic imprinting in the human placenta.** The process involves three main steps – reduced representation bisulfite sequencing of placental samples (red), selection of partially methylated regions with high concordance (green) and individual locus-based validation of the potential DMRs and parental allele-specific expression (violet).

Nine human placental samples (five first trimester and four third trimester) were subject to RRBS analysis for DNA methylation (Figure 1). CpG sites sequenced at greater than 10× coverage were included in the analysis. If our approach was to be used for identifying novel imprinted genes, it should also be able to confirm the known gDMRs. Indeed, CGIs overlapping 14 known human DMRs (known to be gDMRs in mouse) were found to be approximately 50% methylated (Table 1). The DMRs for the genes *MCTS2* and *INPP5F_V2* (described in [57]) were further validated by bisulfite cloning and sequencing and were found to be methylated in an allele-specific manner (Additional file 1: Figure S1). The *NNAT* promoter was not covered by our sequencing data. For the *GNAS* locus, the CGI overlapping with the DMR exhibited 19.1% methylation. However, on analyzing individual CpG sites within this large CGI, the first half of the CGI was found to be about 50% methylated.

### Allele-specific methylation analysis and selection of potential DMRs

**Figure 2:**
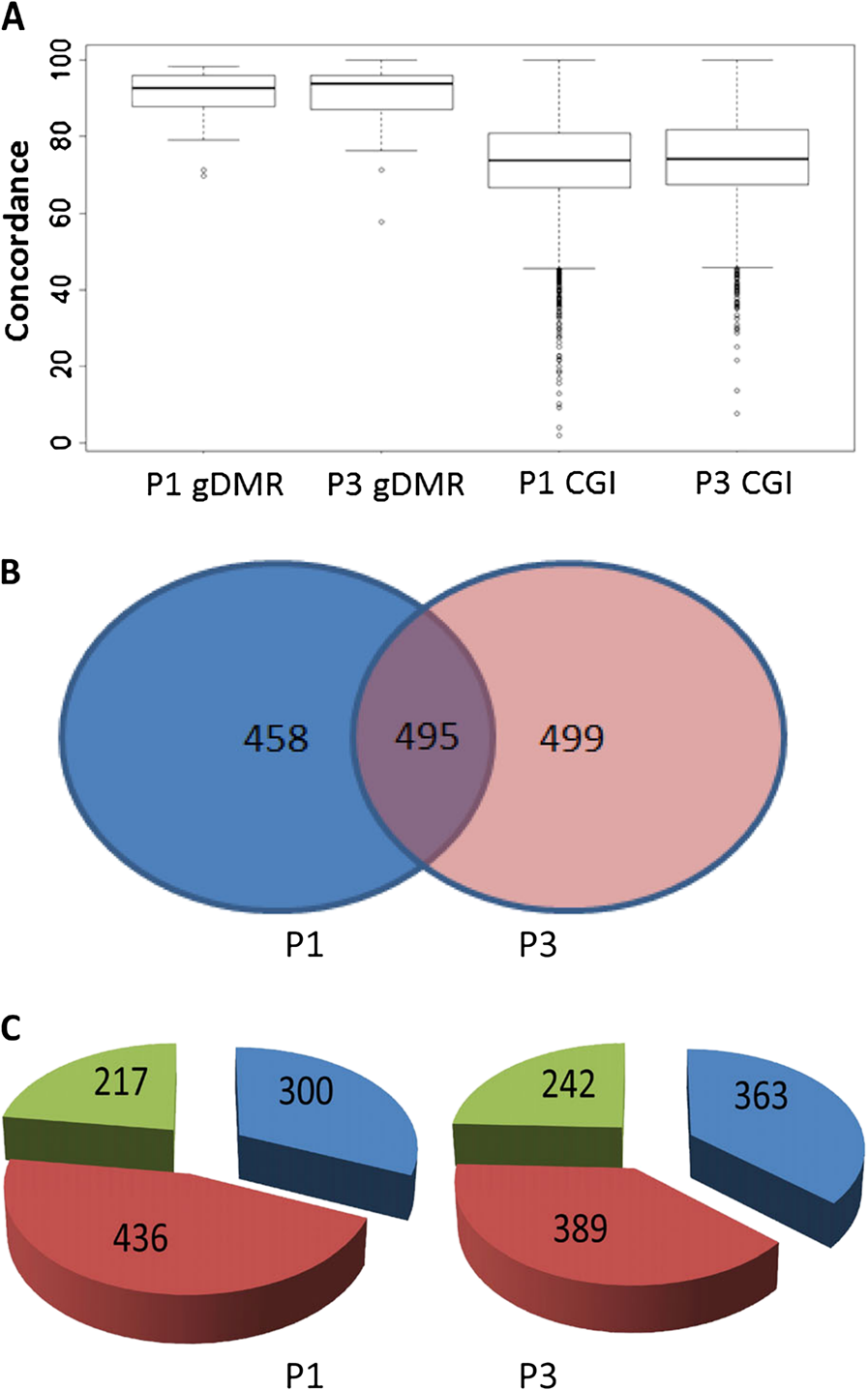
**Methylation concordance in known gDMRs and CGIs with partial methylation. (A)** Comparison of concordance for known gDMRs vs. other CGIs with partial methylation (30-70% methylation): known gDMRs showed much higher concordance levels than partially methylated CGIs. P1: first trimester placenta; P3: third trimester placenta. **(B)** There were 495 CGIs with partial methylation and high concordance shared between the first trimester and third trimester placentas. **(C)** Genomic mapping of CGIs with partial methylation and high concordance showed that the CGIs were distributed across promoters (blue), gene bodies (red) and intergenic regions (green). Gene body regions contained the highest number of such CGIs in both the first and third trimester placenta.

On calculation of a concordance value (see Methods), the known DMRs were shown to be partially methylated with high concordance (Figure 2A). The mean and median concordance values for the first trimester placentas were 90.9% and 92.8% respectively while those for the third trimester placentas were 90.5% and 93.8% respectively. However, other partially methylated CGIs (30-70% methylation) showed a much higher variability in concordance value. We hypothesized that novel DMRs associated with imprinted genes should demonstrate similar methylation patterns to the known DMRs with partial methylation and high concordance.

By choosing partially methylated autosomal CGIs with >85% concordance, we identified 953 regions in first trimester placenta and 994 regions in third trimester placenta, 495 of which were shared between the two trimesters (Figure 2B). These regions were located in promoters, gene bodies and inter-genic regions (Figure 2C). Details of these regions are listed in Additional file 2.

Amongst the 495 potential DMRs overlapping between the first trimester and third trimester placenta samples, we chose 28 genomic regions for further validation. The first 18 regions (Table 2) were chosen based on high expression levels of adjacent genes in placenta as ascertained from RNA-seq data (Jin et al. unpublished data). The other 10 regions (Table 3) were chosen since they were highly methylated in human spermatozoa samples [58] and also had high expression levels of adjacent genes in placenta. These 10 regions were candidates for paternally methylated gDMRs.

**Table 2:** Partially methylated CpG islands with high concordance

Locus*	CpG island*	**Location and annotation**
chr6: 106959764-106960985	CpG 114	Exon-Intron 1 of *AIM1* (melanoma suppressor)
chr7: 807336-808261	CpG 79	Promoter of *HEATR2* alternative transcript
chr19:36604359-36606906	CpG 194	Overlaps Promoters of *TBCB/ POLR21* (polymerase sub-unit gene )
chr19:55992577-55996916	CpG 321	Last exon of *ZNF628*/ promoter of *NAT14* (acetyltransferase)
chr1:41847264-41849204	CpG168	Last exon of *FOXO6*
chr1:111746337-111747303	CpG 94	Promoter *of DENN/MADD* domain containing 2D
chr4:154712073-154712706	CpG 57	Downstream of *SFRP2* (Wnt singnalling)
chr12:22486835-22488666	Cpg 163	Promoter of *ST8SIA1* (sialyltransferase for ganglioside production)
chr13:33001249-33002078	CpG 93	Intron 1 of *NEDD4* binding protein 2-like 1 isoform 1
chr19:1584445-1585247	CpG 89	Exon-intron of *MBD3* (*Nurd* complex subunit: nucleosome remodeling*)*
chr22:29706500_29706710	CpG 15	Exon-intron 3–4 of *GAS2L1* (similar to *Gas2*, Actin-associated protein)
chr9:36,222,678-36,294,377	CpG 76	Promoter of *GNE*, enzyme for N-Acetyl Neuraminic Acid regulation
chr11:497359-511488	CpG 46	Exon-intron 2 of *RNH1* (placental ribonuclease inhibitor)
chr19:10304966-10305864	CpG 89	Promoter of *DNMT1* (DNA Methyl transferase)
chr15:96856299-96875368	CpG 145	Upstream of *NR2F2* (steroid thyroid family of nuclear receptors)
chr2:241496576-241497600	CpG 96	Exon-intron of *ANKMY1* (Ankyrin repeat and MYND domain containing protein)
chr7:127671159-127672853	CpG 156	Exon-intron of *SND1* (p100 co-activator)
chr4:169799086-169799625	CpG 58	Exon-intron of PALLD (cytoskeletal protein involved in actin organization)

*Based on UCSC genome build hg19.

**Table 3:** Partially methylated CpG islands with 100% methylation in sperm

Locus*	CpG island*	**Location and annotation**
chr2:220312699-220314094	CpG 153	Internal exon of *SPEG* (striated muscle protein kinase)
chr3:128215213-128216905	CpG 137	Upstream of *GATA2* (Zinc finger transcription factor)
chr5:343450-344535	CpG 117	Internal exon-intron *AHRR* (aryl hydrocarbon receptor repressor)
chr5:179740711-179741121	CpG 43	Internal exon-intron *GFPT2* (controls flux of glucose into hexosamine pathway)
chr6:1624186-1625468	CpG 111	Promoter of *GMDS* (catalyzes conversion of GDP-mannose to GDP-4-keto-6-deoxymannose)
chr8:145749856-145750410	CpG 61	Promoter of *LRRC14* (Leucine rich repeat containing protein)
chr13:110965775-110966223	Cpg 43	Intron of *COL4A2* (encodes one sub-unit of Type IV collagen)
chr17:46641535-46642110	CpG 56	Intron of *HOXB3* (homeobox transcription factor needed for development)
chr19:36246329-36247982	CpG 127	Promoter of *HSPB6* (heat shock protein, alpha-crystallin related)
chr20:62193967-62198985	CpG 381	Internal exon of *PRIC285* (part of peroxisome proliferator receptor alpha)

*Based on UCSC genome build hg19.

### Analysis of allele-specific expression for genes located in the selected regions in human placenta

**Figure 3:**
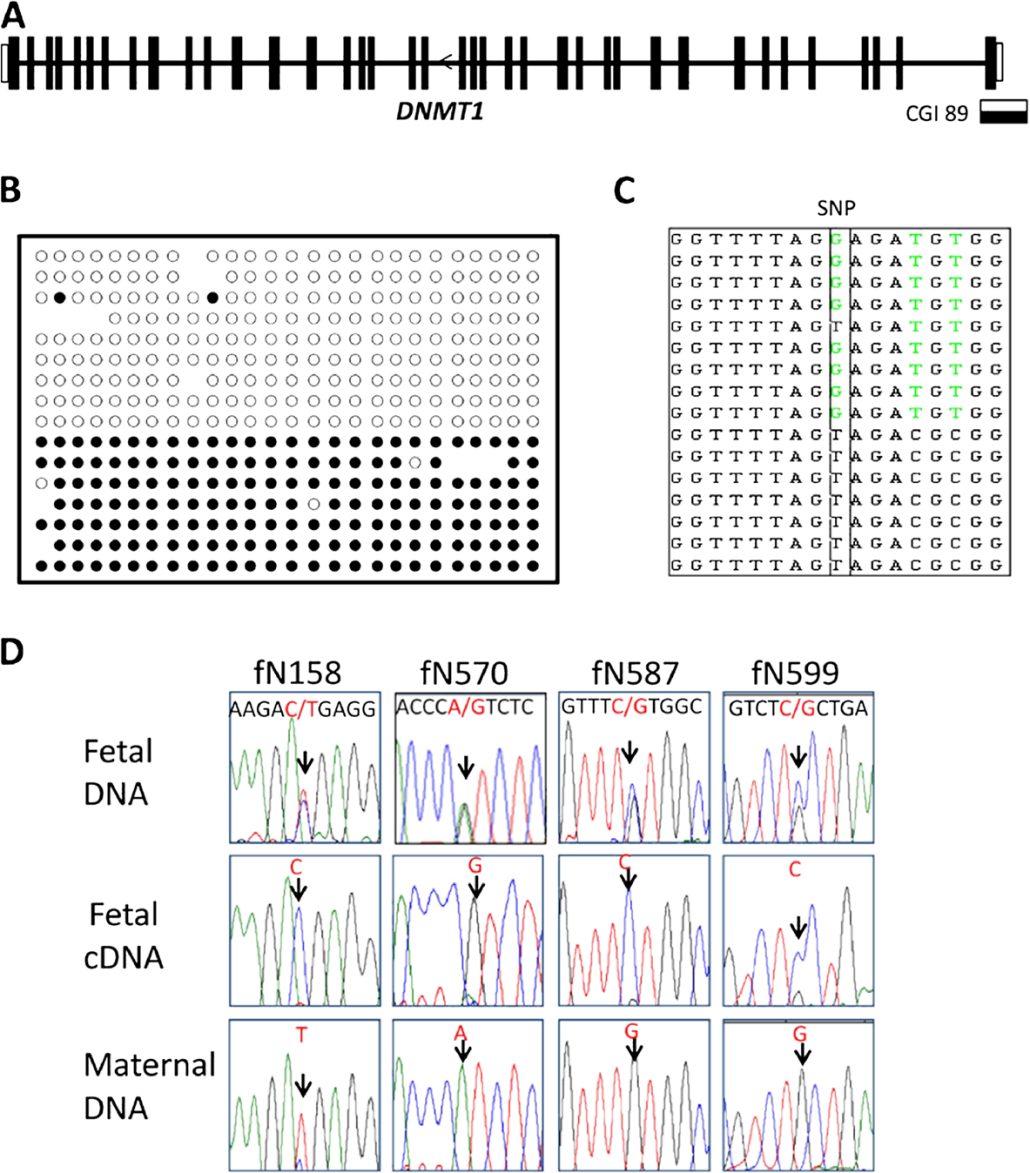
**Methylation and expression analyses of *DNMT1 *in human placenta*. *(A)** CGI 89 is located at the promoter of *DNMT1*. **(B)** Bisulfite treatment and cloning confirmed monoallelic methylation. **(C)** Sequencing of clones from the heterozygous individual fN599 showed that the maternal allele (T allele) was associated with methylated CpGs, while the paternal allele (G allele) was associated with unmethylated CpGs. **(D)***DNMT1* was paternally expressed in humans. Arrow depicts the SNP location for each sample. In all four samples, the fetal DNA was heterozygous and the maternal DNA was homozygous. Fetal RNA showed monoallelic expression from the paternal allele.

**Figure 4:**
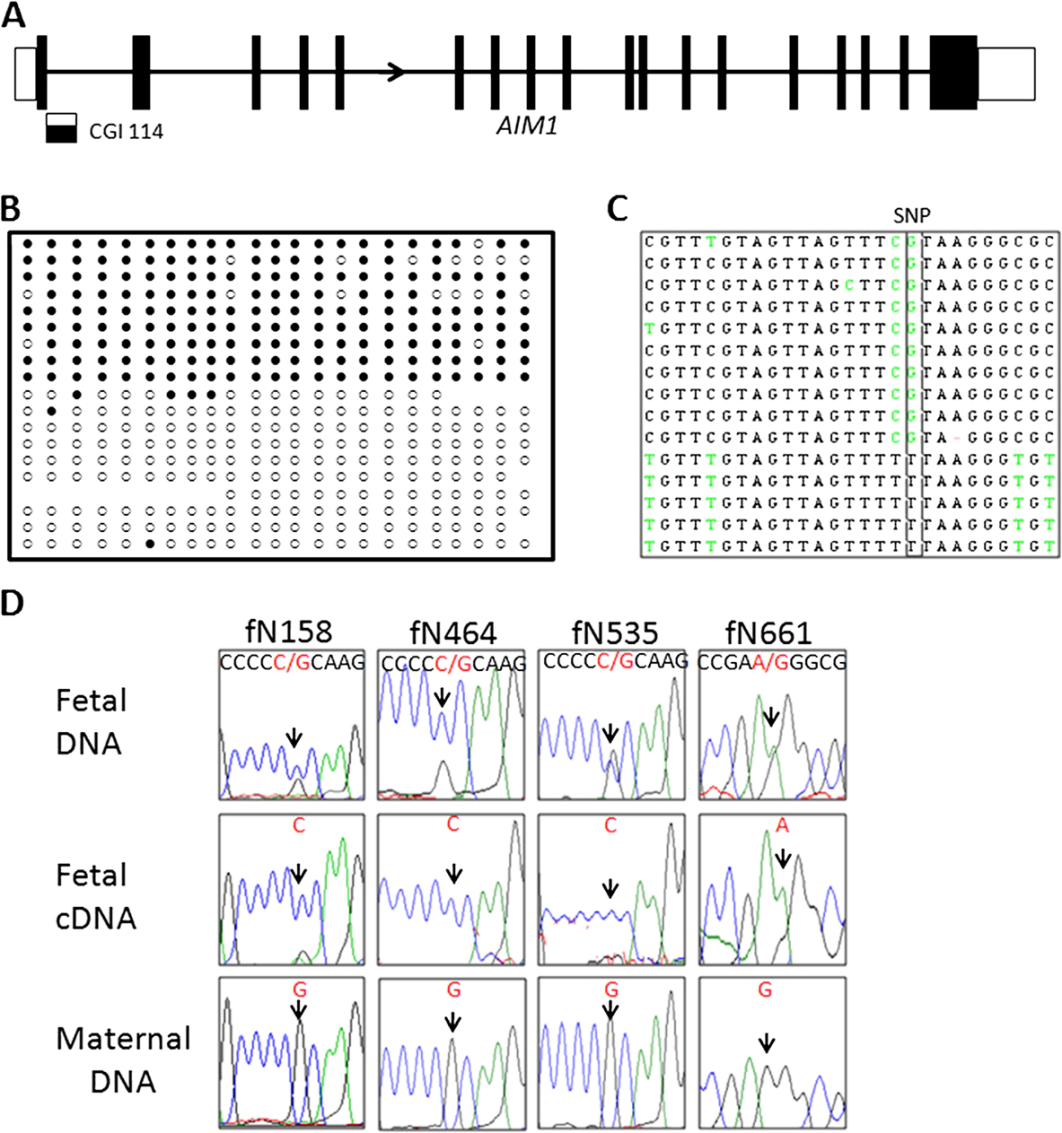
**Methylation and expression analyses of *AIM1 *in human placenta. (A)** CGI 114 is located at the junction of exon 1 and intron 1 of the long transcript of *AIM1*. **(B)** Bisulfite treatment and cloning confirmed about 50% methylation in an allele specific manner in human placenta. **(C)** Sequencing of clones from the heterozygous individual fN158 showed that the maternal allele (G allele) was associated with methylated CpGs, while the paternal allele (T allele) was associated with unmethylated CpGs. **(D)***AIM1* was paternally expressed in humans. Arrow depicts the SNP location for each sample. In all four samples, the fetal DNA was heterozygous and the maternal DNA was homozygous. The fetal RNA showed monoallelic expression from the paternal allele.

We chose 28 genes (Tables 2 and 3) associated with the 28 candidate DMRs for analysis of allele-specific expression. Three to four exonic SNPs per gene were analyzed in 28 paired placental DNA and RNA samples. Two genes (*DNMT1* and *AIM1*) showed allele-specific expression. The monoallelic expression profile was not due to biased expression from one specific allele since reciprocal alleles were represented in the sample set (Figure 3D, Figure 4D, Additional file 3: Figure S3A). For *DNMT1* (or *DNA Methyl Transferase 1*), eight heterozygotes (harboring four SNPs in different exons) exhibited a single allele in their cDNA (four examples are shown in Figure 3D). For *AIM1* (or *Absent in Melanoma 1*), there are two alternative transcripts, the long transcript and the short transcript (Additional file 4: Figure S2). Allele-specific expression was observed in 28 individuals with two different SNPs located in exon 1 specific for the long transcript (four examples are shown Figure 4D). However, bi-allelic expression was observed with SNPs located in exon 20 shared by both the long and the short transcript in two individuals (Additional file 4: Figure S2), indicating that imprinting is limited only to the long transcript of *AIM1*. The bi-allelic status of the shorter transcript was confirmed by 3’ RACE and SNP analysis.

### Methylation and Imprinting analyses of *DNMT1* in human placenta

*DNMT1* has two alternative transcripts, one expressed in somatic tissues (*s-DNMT1*) and the other expressed specifically in the oocyte (*o-DNMT1*). The potential DMR identified at Chromosome 19 CGI 89 is located at the promoter of *s-DNMT1*. We performed bisulfite cloning and sequencing for this region in three human placenta samples to confirm the allele-specific methylation status (one representative example shown in Figure 3B). An individual (fN599) with an informative SNP exhibited methylation of the maternal allele (T) and was unmethylated on the paternal allele (G; Figure 3C). The allele-specific methylation profile was confirmed in one more sample (fN134, Additional file 3: Figure S3B). However, parent-of-origin methylation could not be determined for the second sample since the mother was also heterozygous at this locus.

All the eight polymorphic human placenta samples showed monoallelic expression. Informative SNPs were available in four samples where the mothers were homozygous. All four samples showed paternal allele-specific expression of *s-DNMT1* (Figure 3D).

### Methylation and Imprinting analyses of *AIM1* in human placenta

We performed bisulfite cloning and sequencing for four human placental DNA samples for the Chromosome 6 CpG 114 region (located within *AIM1*). All four samples displayed equal numbers of methylated and unmethylated clones, characteristic of a DMR (one representative example shown in Figure 4B). An individual (fN158) with an informative SNP at the DMR exhibited a methylated maternal allele (G), while the paternal allele (T) was unmethylated (Figure 4C). This maternal allele-specific methylation profile was confirmed in one more individual (fN155, Additional file 3: Figure S3C). We also confirmed that the methylation profile was not a SNP effect by profiling a sample (mN158) that was non-polymorphic at the same locus. This sample still showed the characteristic allele-specific methylation profile (Additional file 3: Figure S3D).

Allele-specific expression was observed in 28 individuals with two different SNPs located in exon 1 specific for the long transcript. Four of the mothers were homozygous at the corresponding SNP loci and thus were informative for parent-of-origin expression analysis. All four placenta samples displayed paternal expression of this gene (Figure 4D).

### Methylation and Imprinting analyses of *AIM1* in cynomolgus macaque placenta

**Figure 5:**
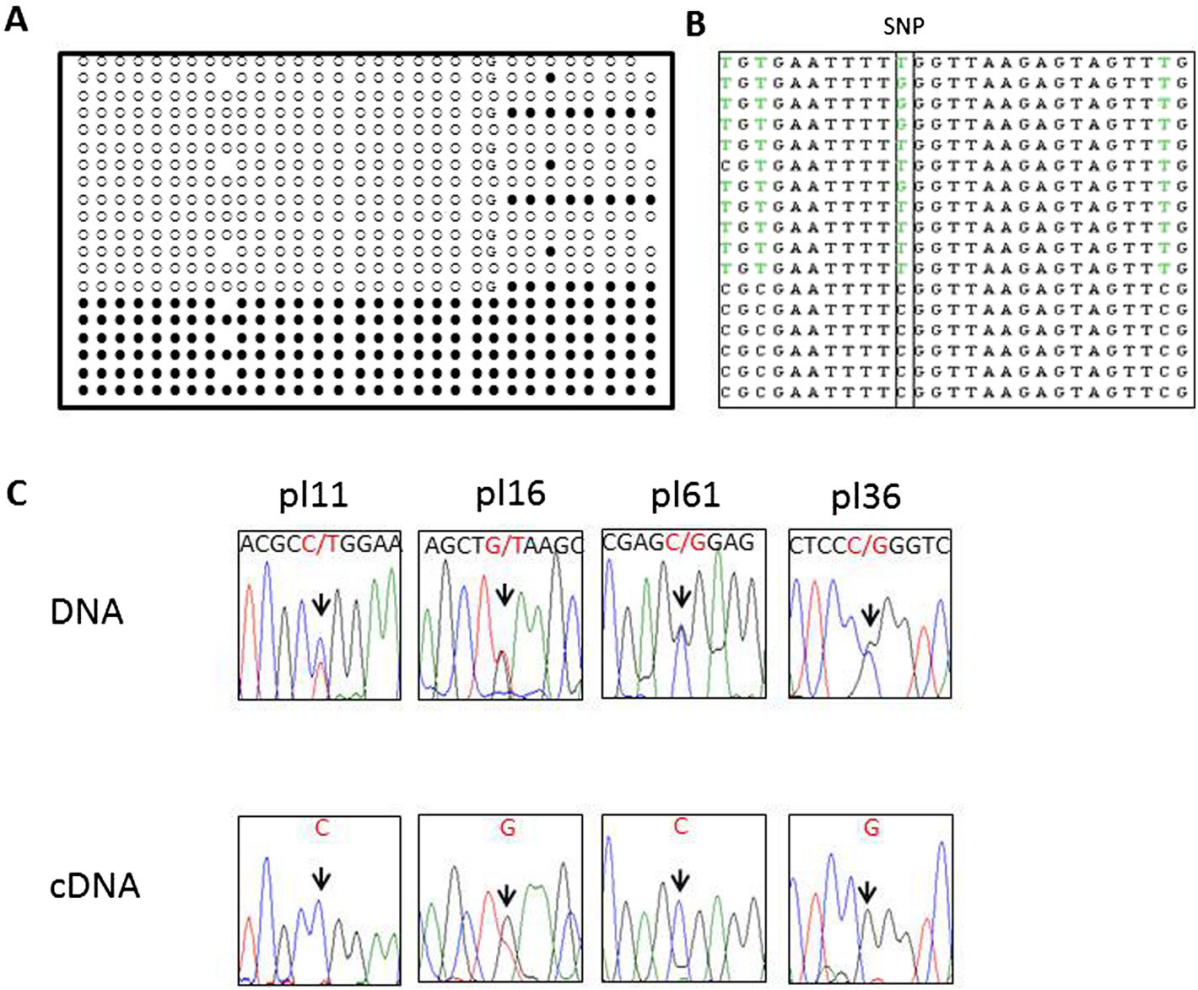
**Methylation and expression analyses of *AIM1 *in macaque placenta. (A)** The region homologous to the *AIM1* DMR in the macaque exhibited approximately 34% methylation. **(B)** Sequencing of clones from the heterozygous individual pl36 showed that the expressed allele (G allele) was associated with unmethylated CpGs, while the non-expressed allele (C allele) was partially methylated. **(C)***AIM1* was monoallelically expressed in the macaque. Arrow depicts the SNP location for each sample. In all four samples, the DNA was heterozygous and the RNA showed monoallelic expression.

The region homologous to human CpG 114 in the macaque was analyzed in the placental DNA of three macaques and shown to have approximately 34% methylation in each sample (one representative example shown in Figure 5A). Analysis of one macaque (pl36) with a C/G polymorphism within the DMR indicated that the expressed allele (G) was completely unmethylated, whereas the non-expressed allele (C) was partially methylated (Figure 5B).

Eleven macaque placental tissues were further analyzed for expression, and four heterozygotes for the first exon were found. Monoallelic expression was observed in all four samples (Figure 5C). Since parent/offspring matched samples were not available, we were unable to determine the parental origin of the expressed allele in these animals.

### Methylation and imprinting analyses of *AIM1* in other cynomolgus macaque tissues

Additional macaque tissues (liver, biceps, kidney, heart, lungs and pancreas) were available for two of the informative individuals, and these were also subject to methylation analysis at the DMR. However, all the tissues were found to be completely unmethylated (Additional file 5: Figure S4). *AIM1* was expressed in the heart, kidney and placenta, but showed minimal or no expression in the liver, lung and pancreas by qPCR analysis (Additional file 6: Figure S5). *AIM1* was found to be bi-allelically expressed in the kidney and heart in two macaques (example of Macaque 11 is shown in Additional file 6: Figure S5).

### Methylation and imprinting analyses of *Aim1* in mice

**Figure 6:**
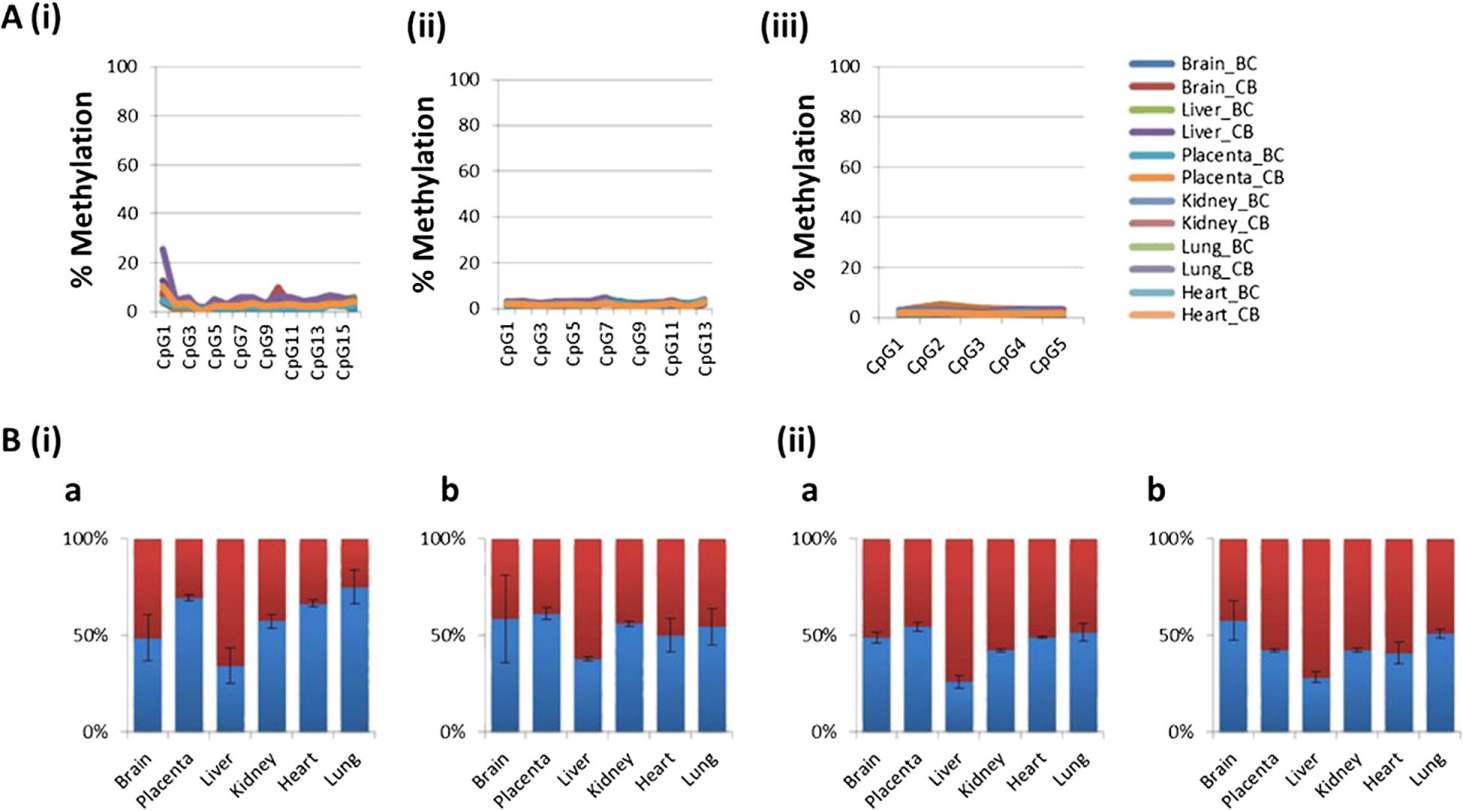
**Methylation and expression analyses of mouse *Aim1*. (A)** Methylation levels in various tissues in the CAST/EiJ X BL6 (CB) and the reciprocal cross BL6 X CAST/EiJ (BC) at both the promoter regions **(i)** and **(ii)** as well as the upstream region **(iii)** were approximately 0-5%. **(B)** Allelic expression of two C/T SNPs in exon 1 **(i)** and exon 2 **(ii)** respectively showed that the gene was bi-allelically expressed in both the BL6 X CAST/EiJ **(a)** and CAST/EiJ X BL6 **(b)** cross. Red bars represent the CAST/EiJ (C) allele whereas blue bars represent the BL6 (T) allele.

Twelve samples from reciprocal crosses of CAST/EiJ and BL6 mice were analyzed for methylation levels in the *Aim1* promoter, and in a second region with a potential alternative transcription start site upstream of *Aim1*. Both regions were hypomethylated in this species (Figure 6A, Additional file 7: Table S3). *Aim1* was expressed in the kidney, placenta and heart but showed minimal expression in the liver, brain and lung (Additional file 8: Figure S6) by qPCR analysis. Bi-allelic expression was observed in all tissues (placenta, brain, heart, liver, lung, and kidney) in the reciprocal crosses (Figure 6B).

## Discussion

The human placenta was chosen for our investigation of novel imprinted genes since genomic imprinting is critical for placenta and embryo development. Additionally, morphological and physiological differences are evident between mouse and human placenta, consistent with differences in imprinting between these two species. RRBS was used to quantify DNA methylation at CpG-rich regions, since it allowed us to readily distinguish two different types of partially methylated regions: those with allele-specific methylation which show high concordance; and those that exhibit variable methylation where different CpGs on the same allele can be methylated or unmethylated [59].

Our DNA methylation data at single base resolution confirmed 16 known DMRs associated with imprinted genes. One known DMR (at the promoter of *NNAT*) was not confirmed because the genomic region was not analyzed by RRBS. As expected, the known DMRs were partially methylated with high concordance. Thus, we selected 28 candidate DMRs from 495 partially methylated regions with high concordance in both first and third trimester placenta samples for analysis of allele-specific expression of adjacent genes. Subsequently, we confirmed that *DNMT1* and *AIM1* were maternally methylated and paternally expressed. While we were preparing the manuscript, a similar theoretical model was used to describe allele-specific methylation in the human genome [60]. The authors identified known imprinted DMRs from publically available methylome datasets in predominantly cultured cells. Another related model has also been used to detect allele-specific methylation in the *Arabidopsis* genome [61].

The Chromosome 19 DMR is located at the promoter of the well-studied *DNMT1* gene. DNMT1 selectively methylates hemi-methylated DNA, regulates tissue-specific methylation and is also essential for maintenance of progenitor cells in an undifferentiated state in somatic tissues [62]. It produces two transcripts, one expressed in somatic tissues (*s-DNMT1*) and the other expressed specifically in the oocyte (*o-DNMT1*). The promoter of *s-DNMT1* was shown to be monoallelically methylated specifically in the primate placenta and to be hypomethylated in other human tissues [63]. Novakovic et al. reported the monoallelic methylation in human placenta to be random, based on only one sample harboring a SNP (rs8112895) in the promoter of *s-DNMT1*. In another recent report, an elegant screen for potential DMRs using diandric and digynic conceptuses also identified the same DMR at the s-*DNMT1* locus, although again only one informative individual was analyzed to confirm monoallelic expression [64]. In our study, we confirmed monoallelic expression in eight individual samples, paternal allele-specific expression in four of these individuals, and maternal specific methylation in one sample with an informative SNP. The latter SNP is located in the CGI within the *DNMT1* promoter (within exon 1, 428-bp away from the polymorphism used in the earlier report [63].

It is interesting to note that the promoter of *s-DNMT1* has already been shown to be monoallelically methylated in the placenta of baboons and marmosets (Figure 4, [48]). Thus, it is likely that placental genomic imprinting of *DNMT1* is maintained throughout the primate lineage. Since we lacked parent-offspring matched samples for our macaque tissues, we were unable to confirm the parental-allele specific expression of *DNMT1* in this species.

It is also interesting that the promoter of *s-DNMT1* has been shown to be unmethylated in the mouse placenta (Figure 4[48]). However, dynamic methylation changes have been observed upstream of the *o-DNMT1* transcript during early mouse development [65]. No evidence for imprinting of murine *Dnmt1* has emerged from genome-wide placenta specific imprinting studies in mice [18,23]. Thus, it appears that genomic imprinting of *DNMT1* is specific to the primate placenta.

The function of the paternal allele-specific expression of *DNMT1* in human placenta remains to be elucidated. Methylation of the *s-DNMT1* promoter may attenuate its transcription; this is coincident with global hypomethylation of the human placenta [63]. Moreover, *s-DNMT1* expression attenuation has been reported to cause alterations in methylation at germline DMRs [66,67]. Thus, it is possible that reduction in *s-DNMT1* level in the human placenta by genomic imprinting is linked to loss-of-imprinting observed at several loci in this tissue [38,68].

*AIM1* or *Absent in Melanoma 1* is a non-lens member of the βγ-crystallin superfamily [69,70]. It was predicted to be a suppressor of malignant melanoma and NK-cell malignancies [71]. It was implicated in trophoblast differentiation in the placenta [72]. It has two alternative transcripts and both are highly expressed in the placenta.

The Chromosome 6 DMR (CpG 114) lies at the exon1-intron1 junction of the long transcript of gene *AIM1*, 460-bp downstream of the transcription start site. There is another CpG island (CpG 44) located 587-bp upstream of the transcription start site of this transcript. CpG 44 is unmethylated. Thus, CpG 114 potentially regulates the expression of the long transcript. The DMR and imprinted expression were found to be conserved in the macaque placenta, but not in the mouse placenta. It should be noted that in the macaque placenta the non-expressed allele was partially methylated while in the human placenta the non-expressed allele was fully methylated. We suspect that this was due to maternal cell contamination in the macaque samples, since the macaque placenta is much thinner than the human placenta, making it difficult to isolate pure fetal cells.

It is interesting to speculate about the function of paternal allele-specific expression of the long transcript of *AIM1*. Since this transcript appears to be robustly expressed in the placenta [70], it is possible that its expression regulated by imprinting is functionally relevant in this tissue. *IGF2R*, one example of a maternally expressed imprinted gene is located on the same chromosome. However, it is unlikely that they belong to the same imprinted cluster since they are approximately 53 Mb apart. Moreover, *IGF2R* exhibits polymorphic imprinting in humans [73].

Limited numbers of validated novel imprinted genes were discovered in previous genome-wide screens, raising the question whether most imprinted genes had been identified [36]. Despite evidence suggesting extensive loss of imprinting in the human placenta [74], our study as well as others [21,37] suggest that novel species and tissue-specific imprinted genes remain to be discovered [4]. The functional consequences of such imprinting events may be species, tissue, and even developmental stage specific. In this regard, the placenta may be a good tissue for studying genomic imprinting since it is both functionally important and evolutionarily under intense selective pressure.

However it is also clear from our data that while allele-specific DNA methylation may be prevalent, most of these epigenetically regulated regions are not associated with genomic imprinting. Out of the 28 potential DMRs analyzed, only two were shown to be imprinted DMRs. We confirmed the allele-specific methylation profile of 10 additional regions by bisulfite cloning and sequencing, even though these were not associated with monoallelic expression. Many of these potential DMRs are located in gene-bodies (Figure 2C and 2D). Some of these regions could contribute to processes like alternative splicing [75], or replication timing [76].

One limitation of our study is the use of RRBS rather than whole genome bisulfite sequencing for the discovery of imprinted genes. RRBS enriches for CpG-rich regions, particularly CpG islands. It is possible that this led to identification of only maternally methylated DMRs in our study, since all known paternally methylated gDMRs are in CpG-poor, inter-genic regions. Analysis of the ten partially methylated regions which were fully methylated in sperm and were potential paternal gDMRs did not yield any imprinted gene candidates. Whole genome bisulfite sequencing analyses would facilitate future discovery of gDMRs in an unbiased manner.

## Conclusions

In conclusion, we have developed a method to study allele-specific methylation and associated genomic imprinting in the human placenta. Careful follow-up and validation of other partially methylated regions with high concordance will potentially reveal the functional role of methylation in these regions and may help identify more novel imprinted genes. The precise functions and mechanisms associated with placenta-specific imprinting of *DNMT1* and *AIM1* remain to be investigated. A more complete catalog of species-specific imprinted genes in the placenta will help our understanding of how genomic imprinting is associated with placental function, morphology and evolution.

## Methods

### Study participants and sample processing

Women with euploidy pregnancies who attended KK Women’s and Children’s Hospital, Singapore, were recruited. Informed consent was obtained under the ethics approval from the SingHealth CRIB Committee (IRB Reference: EC200903042, CIRB Reference: 2009/271/A). Maternal ethnicity was ascertained to be Chinese in four out of the five first trimester placenta cases and Asian for the remaining one. Amongst the four third trimester placenta cases, three were Chinese but the maternal ethnicity for one subject was not available.

Ten mL of peripheral blood from each subject was collected in EDTA tubes. The blood samples were centrifuged at 1,790 g for 10 min at 4°C. After removing the supernatant plasma, the blood cells were transferred to a new microcentrifuge tube and centrifuged at 2,300 g for 5 minutes at room temperature to remove the residual plasma. The blood cells containing buffy coat were then collected and stored at −80°C. DNA was extracted from 200 μL of blood cells using QIAamp DNA Blood Mini Kit (QIAGEN GmbH, Germany), according to manufacturer’s instructions. DNA samples eluted with 50 μL of DNase and RNase-free water (Sigma-Aldrich, St. Louis, MO, USA) were stored at −80°C.

First trimester villi samples were collected from leftover material obtained via chorionic villus sampling. The villi samples were washed extensively with diethylpyrocarbonate (DEPC)-treated water. Third trimester placental villi were collected from subjects after the normal babies were delivered. The fetal side of the placenta was washed with 1×PBS before dissection. A small piece of placenta tissue 1 cm below the chorionic plate was dissected and maternal blood was thoroughly washed away with DEPC-treated water. These samples were stored at −80°C immediately (for DNA extraction), or incubated with RNA*later* (Applied Biosystems/Ambion, Carlsbad, CA, USA) overnight at 4°C, and stored at −80°C (for RNA extraction). DNA and RNA extractions were performed with QIAamp DNA Mini Kit (QIAGEN) and TRIZOL Reagent (Invitrogen, Carlsbad, CA, USA), respectively, according to manufacturer’s instructions.

### Reduced representation bisulfite sequencing (RRBS) and GA analysis

One to five microgram each of genomic DNA from the nine human placental samples was fragmented by restriction enzyme digestion using both Taq^α^I and MspI (New England Biolabs, Ipswich, MA, USA), and was end-repaired, 3’-end-adenylated, and adapter-ligated using ChIP-Seq Sample Preparation Kit (Illumina, San Diego, CA, USA). Illumina’s RRBS for Methylation Analysis protocol was followed, except that 10 μL of the methylation adapter oligo was used and the ligation was performed for 15 min at 20°C in the adapter-ligation step. The gel-purified fragments were then bisulfite treated using the EZ DNA Methylation-Gold Kit (Zymo Research, Irvine, CA, USA), according to manufacturer’s instructions. The converted DNA was PCR enriched, purified by gel electrophoresis, and the library was validated using Agilent 2100 Bioanalyzer (Agilent Technologies, Santa Clara, CA, USA). RRBS was performed on the Illumina Genome Analyzer IIx platform, as per manufacturer’s instructions. The paired-end 36-base pair (bp) reads were filtered based on qseq score, then aligned to CGIs in the bisulfite-converted human genome (UCSC Build hg19, GRCh37, Feb 2009) using Bowtie [77]. The formula for computing bisulfite conversion rate was:

$$\begin{array}{l} \text{Bisulfite}\;\text{Conversion}\;\text{Percentage}\\ \;=\left(\text{nonCpG}\;C‒>T\right)/\left(\text{nonCpG}\;C‒>C+\text{nonCpG}\;C‒>T\right)\\ \;*100\%. \end{array}$$

A bisulfite conversion rate above 99.3% was used as the cut-off.

The formula for computing the CpG methylation for the CpG sites was:

$$\begin{array}{l} \text{CpG}\;\text{Methylation}\;\text{Percentage}\\ \;=\left(\text{CpG}\;C‒>C\right)/\left(\text{CpG}\;C‒>C+\text{CpG}\;C‒>T\right)*100\%. \end{array}$$

The % CpG coverage achieved by RRBS is 3.3%. Genomic regions with at least three CpGs covered at a minimum sequencing depth of 10 were considered. We covered 76.7% of all CGIs.

### Selection of regions with high concordance and partial methylation

The average CGI methylation was obtained by averaging methylation percentage of all the CpGs in a defined CGI. This required that the CGI had at least 3 CpGs with 10× or greater coverage. The CpGs were not necessarily within a limited interval but had to be inside the defined CGIs. CGIs with partial methylation ranging from 30% to 70% were selected. Those with high concordance (>85%) were further short-listed. The concordance of adjacent CpGs in the same read was defined as the ratio of the number of identically methylated (or unmethylated) CpG pairs over the total number of CpG pairs. The average CGI concordance was obtained by averaging concordance of all the CpGs in the defined CGIs. The partially methylated CGIs with concordance >85% were annotated based on whether they belonged to promoters, gene-bodies or inter-genic regions. The promoter of a gene was defined as 1000 bp upstream to 500 bp downstream of the transcription start site for this analysis.

The above analysis was carried out on the first and third trimester placentas separately. Comparing the lists of CGIs with partial methylation and high concordance from the first and third trimester placentas, there was an overlap of 495 CGIs shared between the two.

To assess the possibility of false positives in our dataset of partially methylated genomic regions, we analyzed 37 CGIs known to be unmethylated in first trimester placenta samples [78]. Nine of these 37 CGIs were not analyzed by the RRBS methodology used in this study. The remaining 28 CGIs were almost completely unmethylated in our data (average methylation 2.62%, Additional file 9). None of these 28 CGIs were present amongst the candidate regions identified in our dataset, indicating a low false positive rate.

To shortlist CGIs that are potential gDMRs, MeDIP methylation data for three human spermatozoa samples was downloaded from http://www.ncbi.nlm.nih.gov/geo/query/acc.cgi?acc=GSE25686[58]. Overlapping CGIs between the two data sets were compared to identify regions that exhibited 100% methylation in the sperm but partial methylation with high concordance in the placental samples from our data.

### Imprinting analysis in human placental samples

For assessing monoallelic expression status of the candidate imprinted genes, we first sequenced DNA of exons containing high frequency SNPs (based on dbSNP) for genes listed in Tables 2 and 3. Once heterozygous samples were identified, we tested whether the gene was monoallelically expressed.

Eighty one human placental tissues were chosen for imprint analysis. Thirty ng of purified genomic DNA was used for amplification with the HotStar Taq DNA Polymerase Kit (QIAGEN) with the addition of Q solution (primer sequences are listed in Additional file 7: Table S2). The thermocycling condition was 15 min at 95°C for heat activation, and 45 cycles of 20 sec at 94°C, 30 sec at 60°C and 1 min at 72°C, followed by a 3-min final extension at 72°C. The amplicons were sequenced using BigDye Terminator v3.1 Cycle Sequencing Kit (Applied Biosystems). Reverse transcription was performed using gene-specific reverse primer (AIM1RT1 *for AIM1*) or oligo dT primer (for the rest of the genes, including *DNMT1*) and Superscript III (Invitrogen). The cDNA obtained was amplified using specific primers (listed in Additional file 7: Table S2) and the same thermocycling conditions as above. The PCR products were subsequently sequenced as above. To determine parent-of-origin expression, maternal blood DNA was also sequenced. An informative case was where the mother was homozygous and the fetus was heterozygous.

To increase throughput, Sequenom multiplex genotyping assays were performed on genes listed in Table 3. Sequenom Typer 4.0 (Sequenom, Inc., San Diego, CA, USA) was used to design four multiplex reactions for 30 SNPs within the 10 selected genes. Twenty eight sample sets (fetal placental DNA, buffy coat maternal DNA and fetal placental cDNA) were used at a concentration of 20 ng/μL in 5 μL PCRs (as above) with 0.5 μM primer concentration and thermocycling conditions of 15 min at 94 °C for heat activation, and 45 cycles of 20 sec at 94°C, 30 sec at 56°C and 1 min at 72°C, followed by a 3-min final extension at 72°C. The PCR products were treated with Shrimp Alkaline Phosphatase for 40 minutes at 37°C followed by 5 minutes at 85°C. The extension reaction was performed as per the manufacturer’s instructions, with a 9 μM extension primer concentration. The sample clean-up, spotting on the chip and laser firing were done as per the manufacturer’s instructions and the data was analyzed using the Typer software.

### Methylation analysis by cloning and bisulfite sequencing

Bisulfite conversion with 1 μg of each genomic DNA sample was performed using the EZ DNA Methylation-Gold Kit (Zymo Research), according to manufacturer’s instructions. One twenty-fifth of converted DNA was used for each PCR (primers listed in Additional file 7: Table S1) with HotStar Taq DNA Polymerase Kit (QIAGEN). The thermocycling condition was 15 min at 95°C for heat activation, and 50 cycles of 20 sec at 94°C, 30 sec at 62°C and 1 min at 72°C, followed by a 3-min final extension at 72°C. The PCR product was TA-cloned into pGEM-T Easy vector (Promega, Madison, WI, USA), and the positive clones with inserts were then subjected to PCR amplification using SP6 and T7 primer set from the vector. Sequencing reaction was performed using BigDye Terminator v3.1 Cycle Sequencing Kit (Applied Biosystems).

### Methylation and imprinting analyses of *AIM1* in macaques

Eleven Cynomolgus macaque (*Macaca fascicularis*) neonatal and placental tissues were collected from the Vietnam Primate Breeding and Development Centre. All animal procedures were approved by Nafovanny, subsidiary of the Ministry of Forestry, Vietnam, and performed in accordance with the guidelines set by the national advisory committee for laboratory animal research (NACLAR) of Singapore. All harvested samples were stored at −80°C until analyses.

A CGI homologous to human CpG 114 was identified by BLAT at chr4:102,561,846-102,563,066 on the macaque (*Macaca mulatta*) genome available on the UCSC genome browser. DNA was isolated, bisulfite treated, amplified (primers listed in Additional file 7: Table S1), cloned into TA vector and sequenced as above.

For imprinting analysis, selective genomic regions were amplified from macaque *AIM1* to analyze polymorphisms located at chr4:102,561,811-102,619,896 and Chr4:102,562,698-102,562,816 (primers listed in Additional file 7: Table S2, same thermocycling conditions as above but with annealing temperature 58°C) and sequenced. The expression of the polymorphisms was then analyzed by extracting RNA from placenta of the informative individuals and reverse transcribing (using gene-specific RT primer MacaqueAIM1RT1 or RT2 as appropriate), amplifying with primers listed in Additional file 7: Table S2 and the same thermocycling conditions, and finally sequencing as above.

Additional tissues (liver, biceps, kidney, heart, lungs and pancreas) were available for two of the informative individuals, and these were also subject to methylation analysis at the DMR region.

### Imprinting analysis in mice

Mouse *Aim 1* is located at chr10:43670113–43724652. It is reasonably well-conserved with human *AIM1*, even though it is in reverse orientation unlike its human counterpart. A UCSC-defined CGI is annotated only at its promoter region (chr10:43723295–43724261), but an upstream region at chr10:43,725,821-43,726,151 with elevated H3K4me3 binding could also potentially serve as an alternate promoter. Thus, two bisulfite-pyrosequencing assays were designed to analyze the methylation levels at the promoter CGI as well as one assay for the upstream region (primers listed in Additional file 7: Table S1). DNA from six tissues collected from two reciprocal hybrid (BL6 × CAST/EiJ) embryos at E16.5 stage were bisulfite treated and subject to quantitative CpG pyrosequencing analysis (as per manufacturer’s instructions).

In order to analyze imprinting at this locus, the CAST/EiJ inbred strain crossed with the BL6 which contains polymorphisms in exon 1 and exon 2 of *Aim1* were used*.* RNA was extracted from the above mentioned hybrid embryo tissue material and cDNA was generated. Quantitative real-time PCR was performed to judge the relative levels of expression in the different tissues (brain, placenta, liver, kidney, heart, lung) using primers listed in Additional file 7: Table S2. The resultant PCR product was pyrosequenced (as per manufacturer’s instructions) in order to quantify the relative proportion of each parental allele for the SNP rs46531577 at chr10:43723761 (within exon 1) and SNP rs29356879 at chr10:43717117 (within exon 2).

## Abbreviations

CGI: CpG island; DMR: Differentially methylated region; RRBS: Reduced representation bisulfite sequencing; MeDIP: Methylated DNA immunoprecipitation; DNMT1: *DNA Methyl Transferase 1*; AIM1: *Absent in Melanoma 1.*

## Competing interests

The authors declare that they have no competing interests.

## Authors’ contributions

RD performed the imprinting analysis experiments in humans and macaques and wrote the manuscript. YKL and YCL analyzed the RRBS data. RS performed the mouse experiments. SJ performed the RRBS experiments and aided human sample collection. PYN ran the machines for RRBS. XML collected the human placenta samples. KC collected the macaque samples. GSKY facilitated human sample collection from the clinic. AFS helped design the mouse experiments and aided preparation of the manuscript. DC helped with conceptualization, experimental design and manuscript preparation. All authors read and approved the final manuscript.

## Supplementary Material

Additional file 1: Figure S1 Methylation Analysis of Mouse gDMRs in Human Placental Tissue. Bisulfite cloning and sequencing showed that the promoters of imprinted genes *INPP5Fv2* (A) and *MCTS2* (B) were methylated in an allele-specific manner in human placental tissue. Click here for file

Additional file 2 Lists the allele-specific methylated regions in human placenta. Click here for file

Additional file 3: Figure S3 Confirmation of Allele-specific Expression and Methylation of *DNMT1* and *AIM1*. (A) fN165 expressed the “T” allele in exon 1 of *DNMT1*, reciprocal of the “C” allele expressed at the “C/T” locus of fN158 shown in Figure 3D. Similarly, fN468 expressed the “G” allele in exon 1 of *AIM1*, reciprocal of the “A” allele expressed at the “A/G” locus of fN661 shown in Figure 4D. (B) fN134 was polymorphic within the *DNMT1* DMR; the G allele was unmethylated whereas the A allele was methylated. (C) fN155 harbored a SNP in the *AIM1* DMR; the G/maternal allele was associated with methylated clones whereas the T/paternal allele was associated with unmethylated clones. (D) mN158 was non-polymorphic within the *AIM1* locus and still exhibited an allele-specific methylation profile, indicating that the methylation pattern was not a SNP effect. Click here for file

Additional file 4: Figure S2 *AIM1* Transcripts and Expression Analysis from Different Exons. Human *AIM1* was mono-allelically expressed from exon 1 of Transcript 1 (an asterisk indicates the location of the SNP) but bi-allelic expression was observed by analyzing SNPs in the last exon (which overlaps with Transcript 2, double asterisk indicates the location of the SNP). The transcript information has been obtained from the Ensembl Genome Browser. Click here for file

Additional file 5: Figure S4 Methylation analysis of the *AIM1* DMR in Additional Macaque Tissues. All tissues other than placenta: liver (A), biceps (B), kidney (C), lung (D), heart (E) and pancreas (F) were found to be unmethylated at the DMR locus. Click here for file

Additional file 6: Figure S5 Expression Analysis of *AIM1* in Macaque Tissues. (A) qPCR for *AIM1* in different macaque tissues of one individual (Macaque 11) showed that it is expressed only in placenta, heart and kidney tissues. (B) Macaque *AIM1* is bi-allelically expressed in the heart and kidney tissue of the same individual. Arrow depicts the genomic location of a C/T polymorphism that was still apparent in the cDNA. Click here for file

Additional file 7: Table S1 Primers Used for Methylation Analysis of *DNMT1* and *AIM1*. **Table S2.** Primers Used for Analysis of Imprint Status of *DNMT1* and *AIM1*. **Table S3.** Methylation percentage for individual CpG sites in various tissues in the mouse for *Aim1* promoter and upstream region. Click here for file

Additional file 8: Figure S6 Expression Analysis of *Aim1* in Mouse Tissues. qPCR for *Aim1* in a panel of mouse tissues showed that it is expressed only in placenta, heart and kidney tissues from both Exon 1 (A) and Exon 2 (B). Blue bars represent the BL6 X CAST/EiJ cross allele whereas red bars represent the CAST/EiJ X BL6 reciprocal cross. Click here for file

Additional file 9 **Lists the loci used to assess false positives.** The RRBS and RNA-seq data have been submitted to NCBI Gene Expression Omnibus under accession no. GSE40955. Click here for file
