# Supplementary figures and images for: DNMT1 and AIM1 Imprinting in human placenta revealed through a genome-wide screen for allele-specific DNA methylation
Source: BMC Genomics. 2013 Oct 5;14:685. doi: 10.1186/1471-2164-14-685 (PMC3829101; doi:10.1186/1471-2164-14-685)

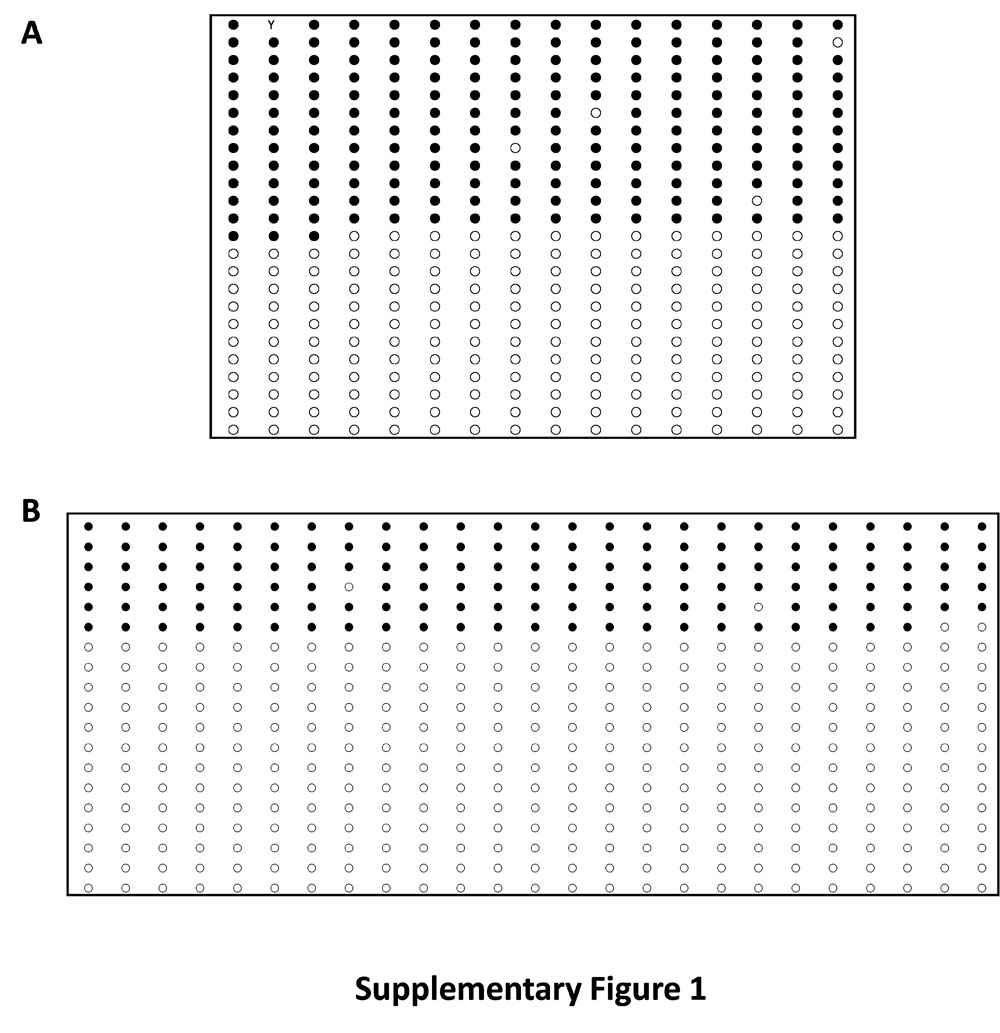

Supplement: Additional file 1: Figure S1 — Methylation Analysis of Mouse gDMRs in Human Placental Tissue. Bisulfite cloning and sequencing showed that the promoters of imprinted genes INPP5Fv2 (A) and MCTS2 (B) were methylated in an allele-specific manner in human placental tissue. [file 1471-2164-14-685-S1.jpeg]

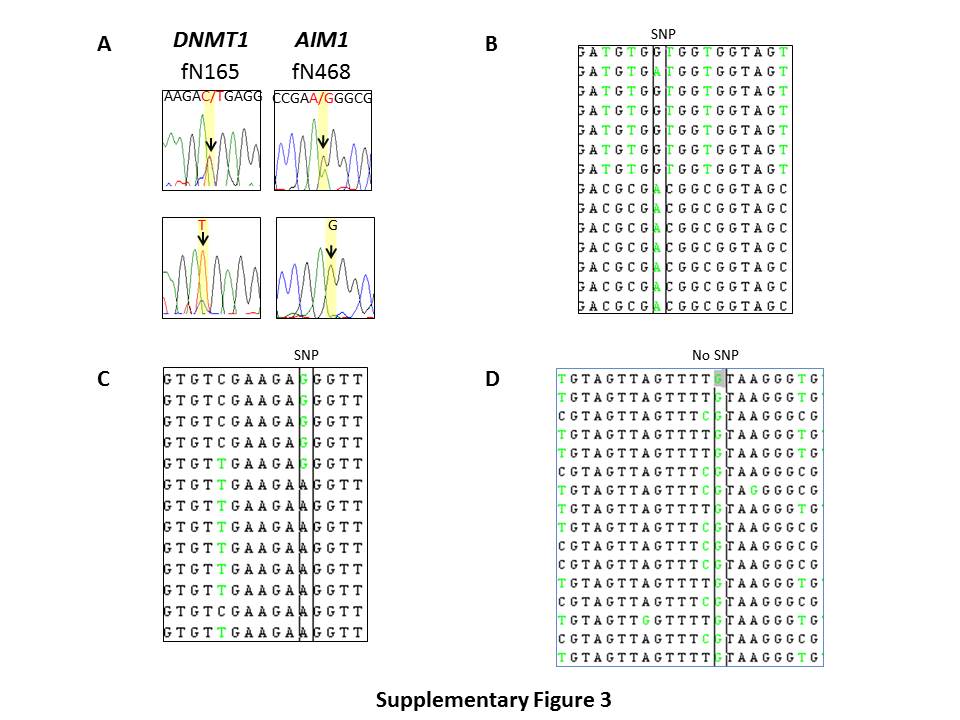

Supplement: Additional file 3: Figure S3 — Confirmation of Allele-specific Expression and Methylation of DNMT1 and AIM1. (A) fN165 expressed the “T” allele in exon 1 of DNMT1, reciprocal of the “C” allele expressed at the “C/T” locus of fN158 shown in Figure 3D. Similarly, fN468 expressed the “G” allele in exon 1 of AIM1, reciprocal of the “A” allele expressed at the “A/G” locus of fN661 shown in Figure 4D. (B) fN134 was polymorphic within the DNMT1 DMR; the G allele was unmethylated whereas the A allele was methylated. (C) fN155 harbored a SNP in the AIM1 DMR; the G/maternal allele was associated with methylated clones whereas the T/paternal allele was associated with unmethylated clones. (D) mN158 was non-polymorphic within the AIM1 locus and still exhibited an allele-specific methylation profile, indicating that the methylation pattern was not a SNP effect. [file 1471-2164-14-685-S3.jpeg]

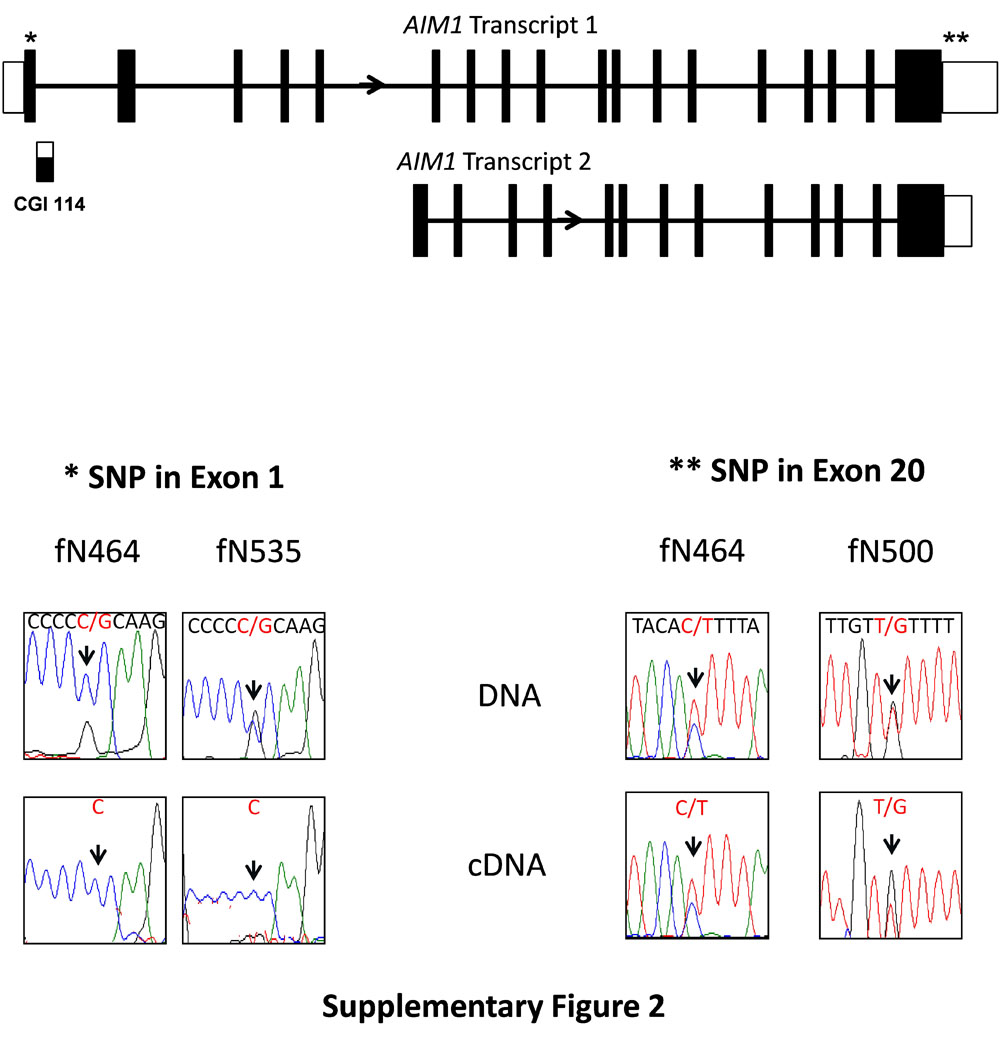

Supplement: Additional file 4: Figure S2 — AIM1 Transcripts and Expression Analysis from Different Exons. Human AIM1 was mono-allelically expressed from exon 1 of Transcript 1 (an asterisk indicates the location of the SNP) but bi-allelic expression was observed by analyzing SNPs in the last exon (which overlaps with Transcript 2, double asterisk indicates the location of the SNP). The transcript information has been obtained from the Ensembl Genome Browser. [file 1471-2164-14-685-S4.jpeg]

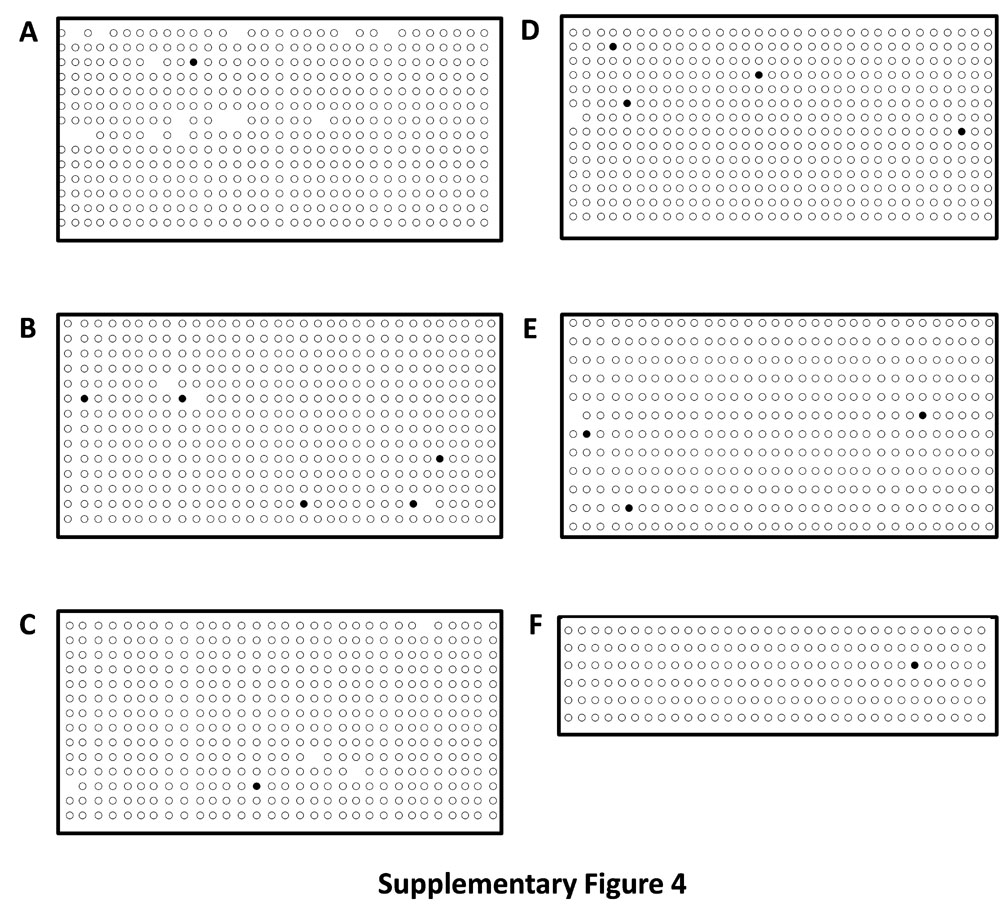

Supplement: Additional file 5: Figure S4 — Methylation analysis of the AIM1 DMR in Additional Macaque Tissues. All tissues other than placenta: liver (A), biceps (B), kidney (C), lung (D), heart (E) and pancreas (F) were found to be unmethylated at the DMR locus. [file 1471-2164-14-685-S5.jpeg]

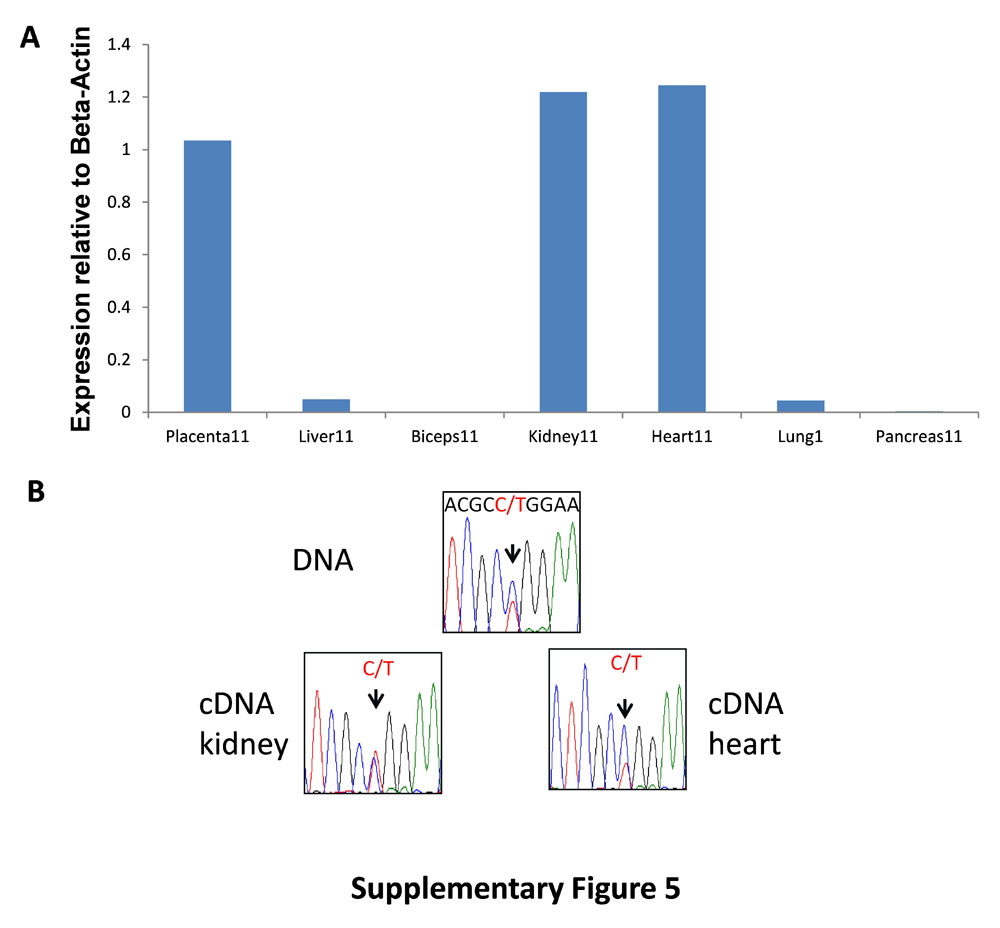

Supplement: Additional file 6: Figure S5 — Expression Analysis of AIM1 in Macaque Tissues. (A) qPCR for AIM1 in different macaque tissues of one individual (Macaque 11) showed that it is expressed only in placenta, heart and kidney tissues. (B) Macaque AIM1 is bi-allelically expressed in the heart and kidney tissue of the same individual. Arrow depicts the genomic location of a C/T polymorphism that was still apparent in the cDNA. [file 1471-2164-14-685-S6.jpeg]

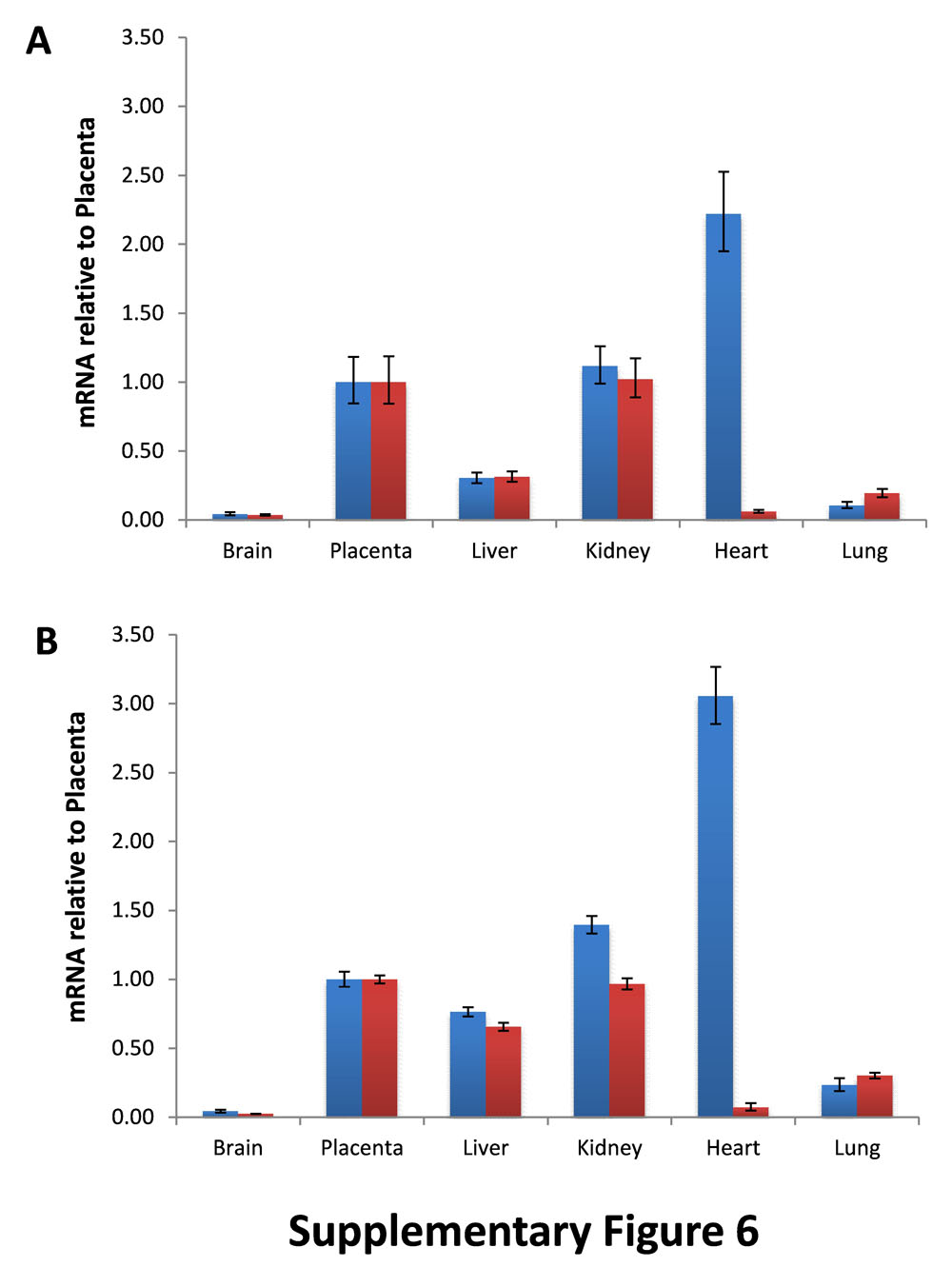

Supplement: Additional file 8: Figure S6 — Expression Analysis of Aim1 in Mouse Tissues. qPCR for Aim1 in a panel of mouse tissues showed that it is expressed only in placenta, heart and kidney tissues from both Exon 1 (A) and Exon 2 (B). Blue bars represent the BL6 X CAST/EiJ cross allele whereas red bars represent the CAST/EiJ X BL6 reciprocal cross. [file 1471-2164-14-685-S8.jpeg]
