# Supplementary material for: DNMT1 and AIM1 Imprinting in human placenta revealed through a genome-wide screen for allele-specific DNA methylation
Source: BMC Genomics. 2013 Oct 5;14:685. doi: 10.1186/1471-2164-14-685 (PMC3829101; doi:10.1186/1471-2164-14-685)
Supplement: Additional file 7: Table S1 — Primers Used for Methylation Analysis of DNMT1 and AIM1. Table S2. Primers Used for Analysis of Imprint Status of DNMT1 and AIM1. Table S3. Methylation percentage for individual CpG sites in various tissues in the mouse for Aim1 promoter and upstream region. [file 1471-2164-14-685-S7.doc]

**Additional file 7: Table S1: Primers Used for Methylation Analysis of *DNMT1* and *AIM1***

| **Oligonucleotide Name** | **Forward Primer** | **Reverse Primer** | **Genomic Location*** | **Size** |
| --- | --- | --- | --- | --- |
| DNMT1bsEx1_F7R7 | TATTGTTGGGTAGTGAGATGGTT | ACTCCATTCCATCCTTCTACAC | 19: 10,305,505-10,305.754 | 249 |
| DNMT1bsEx1_F8R8 | TATCGTCGGGTAGCGAG | GACTCCGTTCCATCCTTC | 19: 10,305,505-10,305,755 | 250 |
| AIM1-hChr6CpG114bs_new2 | GAATAGGAGGTTTTTTTGGGTGT | TTATACCCCCTCAATCTTACTCAAA | 6: 106,959,847-106,960,159 | 312 |
| AIM1-hChr6CpG114bs | GGGAATTAATTGAGGAGGTTGTTAT | CAACCCAATCACCAAAAACACTAC | 6: 106,960,635-106,961,118 | 483 |
| maAIM1_DMR1b | GGAGTAAATAGTTATTTTTGGTTT | ACTCTACCCAACTCTTTACT | 4:102,562,578-102,562,835 | 324 |
| Mus_Aim1_ProReg1 | ATTGTTTGGAGGAAAGGAAAGTGAAAT  Sequencing primer:  GTTTTAGTTAAGGAGTTTTTA | AAAAACCCCTCTTATACTCTAACTTA | 10:43,723,488-43,723,645 | 97 |
| Aim1_ProReg2 | AGTAAAATTGAGGTGGTTTAGGT  Sequencing Primer:  AGGATTAGGAAGATATGGAAAAGAG | CCTCTAAAACAAAAACTAACCTTTAACT | 10:43,723,618-43,723,888 | 270 |
| Aim1_ProUP | GGGGTTGTAGGGTTGGAAAG  Sequencing primer:  AGGGTTGGAAAGGAA | CACTTCTTACCCAAACATTTTACATTTC | 10:43,725,821-43,726,151 | 331 |

* Based on UCSC Genome Browser Build hg19 (for human)/ rheMac2 (for macaque)

**Additional file 7: Table 2: Primers Used for Analysis of Imprint Status of *DNMT1* and *AIM1***

| **Oligonucleotide Name** | **Forward Primer** | **Reverse Primer** | **SNP Location*** |
| --- | --- | --- | --- |
| DNMT1Exon1gDNAF1R1 | CTTGCGCATGCGTGTT | ATGGTACCTACCGCCTGC | chr19:10,305,590 |
| DNMT1Exon1cDNAF1 | GTTCCATCCTTCTGCACA | CCTGCGGACATCGTC | chr19:10,305,590 |
| DNMT1gDNA1 | GCTACTTGCTGTGTATCTGTTC | CAAAGTGCTGGGATTACAG | [rs16999358](http://www.ncbi.nlm.nih.gov/SNP/snp_ref.cgi?type=rs&rs=rs16999358); chr 19: [10273355](http://genome.ucsc.edu/cgi-bin/hgTracks?hgsid=288334775&db=hg19&position=chr19%3A10273355-10273355) |
| DNMT1-RT-1 | CACAGAAGTCAACCCAAAG | GTTTTGCGTCTCTTCTCCTC | [rs16999358](http://www.ncbi.nlm.nih.gov/SNP/snp_ref.cgi?type=rs&rs=rs16999358); chr 19: [10273355](http://genome.ucsc.edu/cgi-bin/hgTracks?hgsid=288334775&db=hg19&position=chr19%3A10273355-10273355) |
| DNMT1gDNA3 | CATTCTCTCATTGCCTCGT | TGCTAGGATTACAGATGTGAGC | [rs2228611](http://www.ncbi.nlm.nih.gov/SNP/snp_ref.cgi?type=rs&rs=rs2228611); chr 19: [10267077](http://genome.ucsc.edu/cgi-bin/hgTracks?hgsid=288334775&db=hg19&position=chr19%3A10267077-10267077) |
| DNMT1-RT-3 | TGTGTACTGTAAGCACGGTC | CCAGTGATCCACCATTCA | [rs2228611](http://www.ncbi.nlm.nih.gov/SNP/snp_ref.cgi?type=rs&rs=rs2228611); chr 19: [10267077](http://genome.ucsc.edu/cgi-bin/hgTracks?hgsid=288334775&db=hg19&position=chr19%3A10267077-10267077) |
| DNMT1gDNA5 | ATGTCTGTTACTCGCCTGTC | AAGAGCTTGTTCCTGTCTGA | chr19:10,244,232  chr19:10,244,069 |
| DNMT1-RT-5 | TGCTAAGGACTAGTTCTGCC | TGGTTTATAGGAGAGATTTATTTG | chr19:10,244,232  chr19:10,244,069 |
| hChr6CpG114_DNA1 | GAAATTCCTCCAGGCAAG | GAGTCGGTGGATTTCTGC | rs1340623;chr 6:106959999 rs4945755; chr 6:106960039 |
| AIM1RT1 |  | TAGATGTCCAAAGTTAGC | - |
| AIM1ex1 | AGGCTTTCCTGGGTGTG | GCTGTGCAGTCTGCCTG | rs1340623;chr 6:106959999 rs4945755; chr 6:106960039 |
| MacaqueAIM1_DNA1 | CTCATATGTTCCCTCCTCAT | CGTCTCTGCGTTCTCTTG | chr4: 102561598 /  chr4:102561616 /  chr4: 102561716 |
| MacaqueAIM1RT1 |  | ATTTCTGCGACCTCCTC | - |
| MacaqueAIM1RT2 |  | TAGTGGTCACCGTCGAC |  |
| MacaqueAIM1cDNA1 | GCCTCTTCTAATCTCCCAGA | GTGAATCATTTTCATGGCAC | Chr 4: 102561598/  chr4:102561616/  chr4: 102561716 |
| MacaqueAIM1cDNA3 | CGAAGAGGGTGCCAGAT | CTCCGACCTCCCTCTG | Chr4: 102,562,788 |
| Mus_Aim1_Exon1 | GGAAGTCGCAGAAATCCACC  Sequencing primer :  CACCTCGTCGTCGAA | CCTTTCCGCCAAGCAGTTCT | rs46531577; chr10:43723761 |
| Mus_Aim1_Exon2 | TTTGTGGGTACTTTTCCATGTG  Sequencing primer :  CATGTGGCTGGAACCC | CAGACAAAGAACAGCCACACCT | [rs29356879](http://www.ncbi.nlm.nih.gov/SNP/snp_ref.cgi?type=rs&rs=rs29356879); [chr10:43717117](http://genome.ucsc.edu/cgi-bin/hgTracks?hgsid=244388377&db=mm9&position=chr10%3A43717117-43717117) |

* Based on UCSC Genome Browser Build hg19 (for human)/ rheMac2 (for macaque)/ mm9 (for mouse)

**Additional file 7: Table 3**

Methylation percentage for individual CpG sites in various tissues in the mouse for *Aim1* promoter and upstream region

Two crosses were used for analysis: the CAST/EiJ X BL6 (CB) cross and the reciprocal cross BL6 X CAST/EiJ (BC). The promoter region was analyzed in two assays (1) and (2). A third assay was used for the upstream region.
